# Worldwide Research Trends in Landslide Science

**DOI:** 10.3390/ijerph18189445

**Published:** 2021-09-07

**Authors:** Paúl Carrión-Mero, Néstor Montalván-Burbano, Fernando Morante-Carballo, Adolfo Quesada-Román, Boris Apolo-Masache

**Affiliations:** 1 Centro de Investigaciones y Proyectos Aplicados a las Ciencias de la Tierra (CIPAT), Campus Gustavo Galindo, ESPOL Polytechnic University, Km 30.5 Vía Perimetral, Guayaquil P.O. Box 09-01-5863, Ecuador; nmontalv@espol.edu.ec (N.M.-B.); fmorante@espol.edu.ec (F.M.-C.); 2 Facultad de Ingeniería en Ciencias de la Tierra, Campus Gustavo Galindo, ESPOL Polytechnic University, Km 30.5 Vía Perimetral, Guayaquil P.O. Box 09-01-5863, Ecuador; 3 Department of Economy and Business, University of Almería, Ctra. Sacramento s/n, 04120 La Cañada de San Urbano, Spain; 4 Facultad de Ciencias Naturales y Matemáticas (FCNM), Campus Gustavo Galindo, ESPOL Polytechnic University, Km. 30.5 Vía Perimetral, Guayaquil P.O. Box 09-01-5863, Ecuador; 5 Geo-Recursos y Aplicaciones (GIGA), Campus Gustavo Galindo, ESPOL Polytechnic University, Km. 30.5 Vía Perimetral, Guayaquil P.O. Box 09-01-5863, Ecuador; 6 Department of Geography, University of Costa Rica, San José 2060, Costa Rica; adolfo.quesadaroman@ucr.ac.cr

**Keywords:** landslides, bibliometric analysis, co-citation analysis, science mapping

## Abstract

Landslides are generated by natural causes and by human action, causing various geomorphological changes as well as physical and socioeconomic loss of the environment and human life. The study, characterization and implementation of techniques are essential to reduce land vulnerability, different socioeconomic sector susceptibility and actions to guarantee better slope stability with a significant positive impact on society. The aim of this work is the bibliometric analysis of the different types of landslides that the United States Geological Survey (USGS) emphasizes, through the SCOPUS database and the VOSviewer software version 1.6.17, for the analysis of their structure, scientific production, and the close relationship with several scientific fields and its trends. The methodology focuses on: (i) search criteria; (ii) data extraction and cleaning; (iii) generation of graphs and bibliometric mapping; and (iv) analysis of results and possible trends. The study and analysis of landslides are in a period of exponential growth, focusing mainly on techniques and solutions for the stabilization, prevention, and categorization of the most susceptible hillslope sectors. Therefore, this research field has the full collaboration of various authors and places a significant focus on the conceptual evolution of the landslide science.

## 1. Introduction

Landslides are disasters that cause damage to anthropic activities and innumerable loss of human life globally [1]. Mass movement processes cause significant changes in the Earth’s relief, causing economic losses due to landslides in mountainous areas with a dense population [2,3], and even in the direct and indirect cost of buildings or infrastructure on an urban scale [4,5,6].

In the evolution of the reliefs, landslides are considered to be intrinsic processes, and among other dynamics, they favor the formation of valleys [7], and the contribution of river sediments and ecological renewal. The degree of physical, biological and chemical weathering, earthquakes, and extraordinary rains (among other natural processes) can cause slope instability [8,9].

Landslides have caused costly damage and loss of life worldwide, yet the most devastating disasters occur in developing countries [10]. Therefore, the implementation of techniques to reduce geological risks and natural vulnerability is essential for developing disaster prevention and mitigation strategies on various scales [11,12,13,14].

This research field has different approaches and objectives that have evolved over the last decades [15]. Some studies have been based on satellite images in remote sensing [16], geomorphological mapping [17,18], its relationship with earthquakes [9], continuous monitoring of places susceptible to landslides [19,20], triggering of landslides due to extraordinary precipitation events [21,22,23] and various methods for stabilizing slopes [24,25].

There are other studies of a preventive nature, such as real-time warnings of landslides due to the action of rains in winter [26] and in unsaturated areas above the water table [27], which are of great support for adequate management of these disasters. The consequences caused by landslides (centralized in an environmental and socioeconomic framework) show that their impacts have greater intensity in areas with higher population density [28]. Across the world, there is a great number of landslides that have affected the population from cold, temperate and tropical regions [13,29,30,31,32,33,34,35].

**Figure 1:**
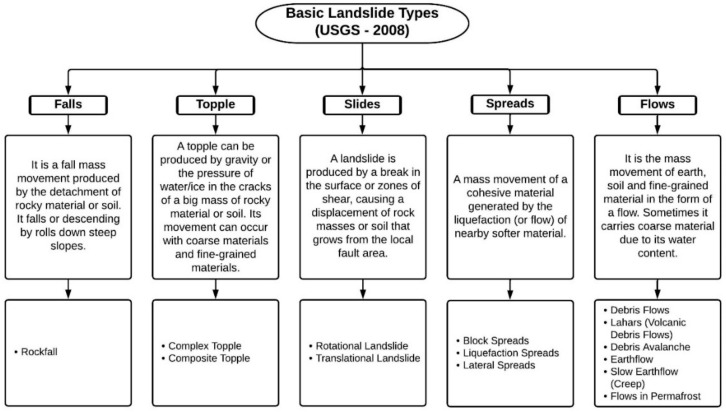
Classification scheme based on the literature review of the USGS landslide manual. Source: [36].

According to the United States Geological Survey (USGS), the material involved in a landslide and its type of mass movement is a significant basis for the classification of landslides [36]. Therefore, given the internal mechanics that predominates in mass movements, the landslides are classified as: falls, topples, slides, spreads, and flows (Figure 1).

The academic field of landslides is broad, where some researchers have made efforts to understand their structure [37], addressing literature reviews [11] and their classification [36,38,39], as well as the bibliometric analysis of various landslide concepts through the Science Citation Index-Expanded (SCIE) and Social Sciences Citation Index (SSCI) databases (1991–2014) [13]. Over time, various studies have been carried out regarding landslides, but very few have highlighted their structure and intellectual growth. Therefore, a new bibliometric study would allow a new approach to its structure and updates on its different research scopes.

The use of bibliometric methods is considered for the analysis of scientific activity in an academic field. Derek J. de Solla Price initially exhibited the bibliometric analysis in 1965 [40]. The proposal focuses on the quantitative evaluation of an academic field of study by analyzing its structure, characteristics and existing relationships, which allows examining its behaviour between the disciplines of a specific field of study [41,42]. The bibliometric analysis allows identifying research areas (current and future) and the analysis of their multidisciplinary production, achieving a more systematic comprehensive evaluation in the field of study [43,44].

Due to the above, the research question arises: How has the intellectual/conceptual structure of the various types of landslides developed over time?

The present study aims to evaluate the intellectual structure of the landslide through performance analysis and bibliometric mapping to determine the development, patterns and trends of its scientific structure. Thus, to analyze the scientific production and intellectual structure of the field of study, managing to provide a transparent, updated, reliable and high-quality study for its transdisciplinary use.

This study has been structured in five sections, starting with an introductory framework of the problem, highlighting its objective and investigative question to support at the end of this work, followed by Section 2, in which the materials and the implemented methodology are described (three phases: research criteria and source identification, software and data extraction, and data analysis and interpretation). Section 3 represents the results and their analysis, to later be discussed in Section 4 and, finally, Section 5 concludes with the scientific trends of this research field.

## 2. Materials and Methods

A systematic review allows an exploration of the intellectual territory of existing studies in the face of a problem raised, evaluating the contributions and synthesizing the data obtained to provide reliable knowledge of a particular field of study [45,46]. This exhaustive and rigorous procedure is similar to the protocol presented in the bibliometric analysis [47,48].

The bibliometric analysis allows evaluating the scientific production of journals [49,50] or understanding the intellectual structure of various fields of knowledge such as management [51,52,53], environment [54,55,56], natural science [57] and health [58]. Employing analytical techniques that allow an exploration of the tendencies of investigation and interpretation of new perspectives in the investigative field [59,60].

**Figure 2:**
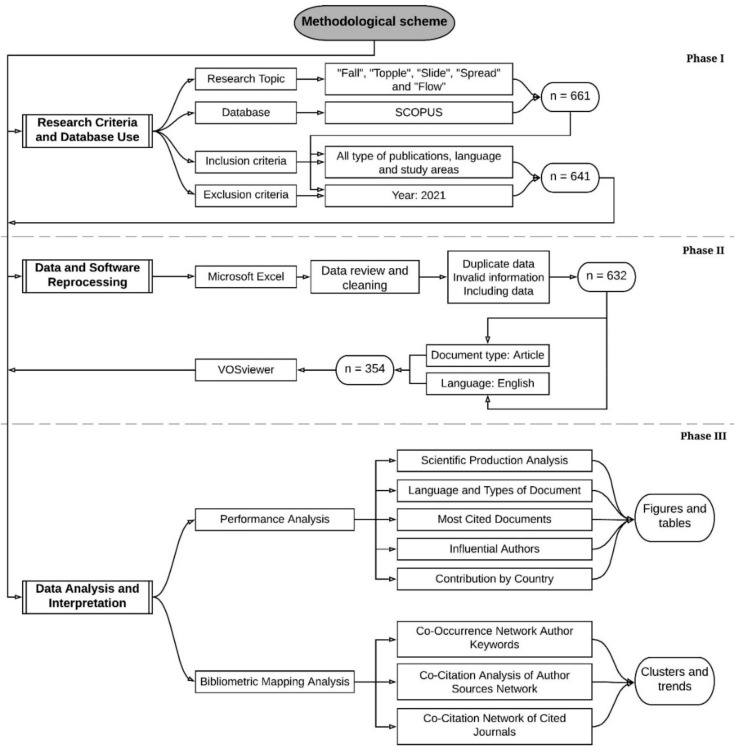
Bibliometric research methodology applied in this study.

The methodology proposed in this work is shown in Figure 2. Its structure comprises three phases that allow the proposed bibliometric analysis to be carried out: (i) Research criteria; (ii) reprocessing of data and software; and (iii) analysis and interpretation of data.

### 2.1. Phase I. Research Criteria and Database Use

For this research, a bibliographic search of the classification of landslides was established based on the internal mechanics of the mass movement. These requirements are encompassed by the USGS, which establishes a classification according to the internal mechanics present in landslides, such as fall, topple, slide, spread and flow [36]. The selection of these terms allows the compilation of the base documents to be considered in this study.

The selection of documents should be made based on choosing a reliable, quality database with comprehensive coverage. The databases used for bibliometric studies are the Web of Science and Scopus, which differ in volume of information, journal coverage and subject areas [61]. The Scopus database was selected due to its comprehensive coverage in years, journals in various areas of knowledge [62,63,64,65], an intuitive search system, easy data download and high-quality standards [66,67], which allows a more precise bibliometric evaluation in the domain of any subject to be analyzed.

The search carried out in Scopus focuses on the titles of the publications that contain the term “landslide” with the terms of: fall, fall, slide, spread and flow. The search topic is as follows: (TITLE (fall*) OR TITLE (topple*) OR TITLE (slide*) OR TITLE (spread*) OR TITLE (flow*) AND TITLE (landslide*)).

The landslide research field is vast, so it is necessary to obtain more exact results and synthesize the study approach; therefore, the search in Scopus focuses only on the title of the publications with the previously established terms [68,69]. In this way, a total of 661 publications were obtained, to which inclusion criteria such as all types of document, language, years and study area were applied [13], in addition to an exclusion criterion such as the year 2021 (year still in progress), obtaining a final database of 641 documents.

### 2.2. Phase II. Data and Software Reprocessing

The selected records are downloaded in csv format (comma separated values) from the Scopus database for analysis using the Microsoft Excel software from Office 365 ProPlus [70]. Since the downloaded database contains miles of data from various variables (e.g., authors name, document title, year, keywords, abstracts, among others), a review and cleaning of the data is required to ensure precision in analysis results [71,72]. Cleaning consists of eliminating duplicated values, incomplete or erroneous records that cannot be completed manually [73]. A total of 9 deleted records and 632 documents to be analyzed were established.

The new csv files were entered in VOSviewer, an open access and reliable software that allows the construction and visualization of bibliometric networks in various fields of study, allowing a comprehensive bibliometric mapping in any research branch [74,75]. This software allows an analysis of the structure of the research field through co-occurrence [76], co-citations [77,78,79,80], and bibliographic coupling [81]. This software has been used in different scientific areas such as: sustainability [82], natural and cultural resources [83], geosciences [55,84], medicine [76] and the circular economy [85], among others. Its analysis is carried out only for articles in English, obtaining a total of 354 documents.

### 2.3. Phase III. Data Analysis and Interpretation

The results were examined using the two classic approaches to bibliometric analysis: Performance Analysis and Science Mapping [42,86].

- Performance analysis allows an evaluation of its scientific production (authors, countries, journals) and its scientific impact [87,88];
- sciences mapping allows the graphic representation of the cognitive structure of the study field and its evolution [41,89]. It is considered to apply a triangulation method that allows an analysis of this structure by examining its micro (keywords), meso (articles and authors) and macro (journals) components [90].

## 3. Results

### 3.1. Performance Analysis

#### 3.1.1. Scientific Production

**Figure 3:**
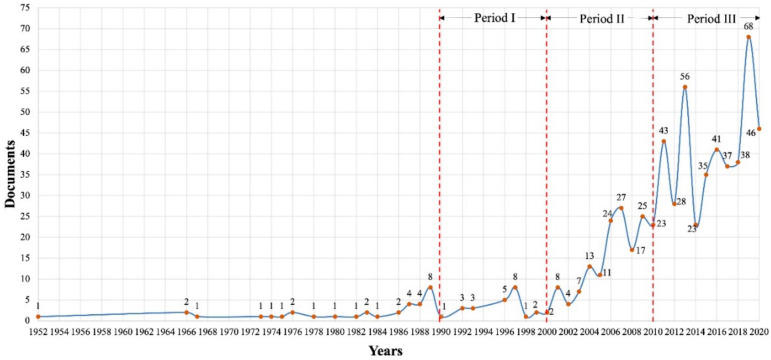
Growth of scientific production of landslides.

From 1952 to 1990 (Figure 3), landslides have been analyzed from a descriptive perspective, considering the internal mechanics and the mass movement type that is generated according to the lithology and the material involved [91,92,93]. Its leading causes are determined, such as the hydraulic gradient and earthquakes [94,95,96,97]. There is also the beginning of geotechnical and geomorphological studies and the elaboration of models to understand the internal mechanics of the different triggered landslides [93,98,99]. Given this analysis, this period is considered to be the beginning of studies that will be the basis for further research.

Figure 3 shows a progressive growth in 1990–2020, determining three different periods that frame the studies.

Period I (1990–2000) focuses on researches related to the debris flows, managing to generate models for the understanding and prediction of landslides, and the volume of material deposited in a sector [100,101]. It considers different aspects such as the mechanical process of mass movement [102,103], data in the field (rainfall, vegetation cover, slope inclination, distance, elevation), coefficient of internal friction, among others [104,105,106,107]. This period is the basis for continuous studies and analysis of future landslide models.

In period II (2001–2010), the exponential research growth and a significant focus on the classification of landslides is observed. These classifications focus on the area of engineering and speed of landslide for the elaboration of physical models [108], considering the material involved (gravel, sand, silt and clay) and its variations (debris, earth and mud, peat and rock), thus managing to formalize definitions that allow identifying the present types of landslides [109,110,111,112]. In 2008, a relevant study to the global analysis of rainfall was presented, which made it possible to study rainfall and its influence on shallow landslides and debris flows [113]. These studies are the basis of all landslide warning systems throughout the world [114,115,116]. From this, the mathematical prediction models have been considered of great importance worldwide, calculating and predicting the trajectory, speed and depth that landslides would have [117,118,119].

Finally, period III (2011–2020) focuses on the improvement and combination of different numerical models, managing to represent the reality of the environment and the mechanical behavior of the landslides for their respective analysis in field and risk assessment [120,121,122,123]. In this way, at the end of this period, these investigations and improved models allow us to understand the behavior of different landslides types [124,125,126]. In addition, the geomorphological, tectonic and hydrodynamic processes involved in mass movement processes were explained in detail [127,128]. Different experimental research was conducted considering the pressure of the pore fluid, type of grain, rainfall and a large amount of on-site and laboratory investigations, assuring the validity of the results [129,130,131,132,133,134].

#### 3.1.2. Language and Types of Documents

In the areas of knowledge related to Life Science and Earth Science, the English language is predominant [135]. Landslide is no exception; despite presenting studies in 15 languages, 81.8% of its studies are written in English. This predilection for language is due to its relevance in scientific communication as there is an overrepresentation of English-speaking journals, and it is the common nexus for international collaboration [136,137]. The second language is Chinese (13.45%), due to its high national collaboration on topics of debris flow and flow-type landslides in national indexed journals (e.g., Yantu Lixue/Rock and Soil Mechanics, Yanshilixue Yu Gongcheng Xuebao/Chinese Journal of Rock Mechanics and Engineering, Journal of Natural Disasters).

**Figure 4:**
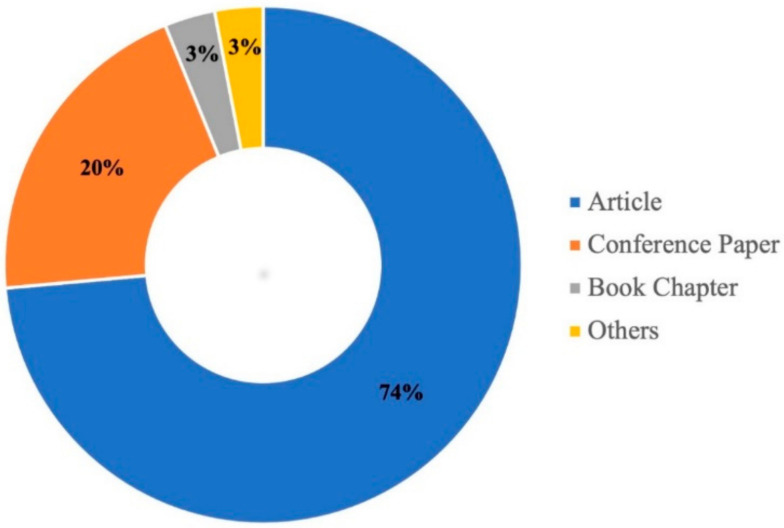
Types of scientific publications.

Another characteristic of landslide studies is that they mostly constitute journal articles (74%) since these documents are considered certified knowledge, as they are examined by peer reviewers who have expertise in the field of knowledge [138]. Other types of documents are shown in Figure 4.

#### 3.1.3. Contribution by Country

**Figure 5:**
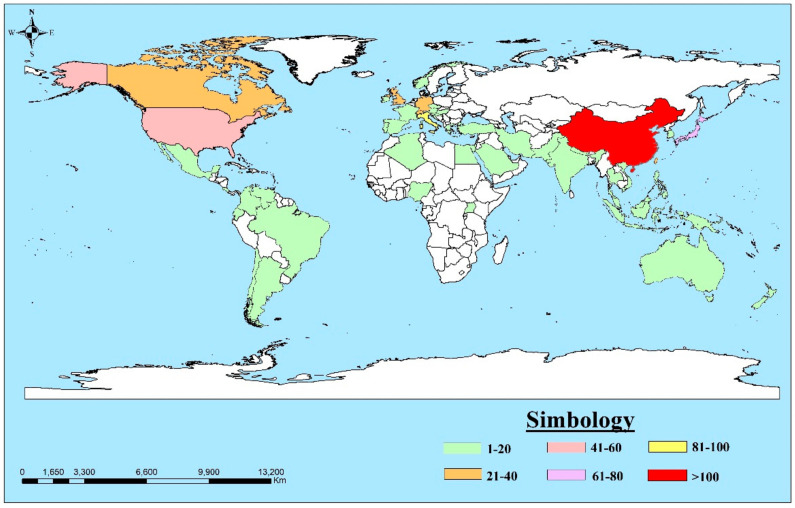
Contribution by countries, world map.

The analysis of the contribution of the countries allows us to understand their relationships in knowledge generation [87]. This product is developed by the collaboration of 64 countries (see Figure 5), in which most of the research is related to developed countries. The map was generated through ArcMap 10.5 software, using data from the authors’ affiliations.

China has the most significant academic contribution on landslides (Figure 5), collaborating with 47 countries, especially Italy, the United Kingdom and the United States. The contributions with Italy are related to numerical modelling in the propagation of flow-like landslides [139,140,141]. Concerning the United Kingdom, studies focus on modelling debris flow and submarine landslides and as a flow influenced by precipitation, earthquakes, or tectonic movements, e.g., [142,143,144]. The third international partner, the United States, focuses on landslide monitoring and numerical modelling based on the smoothed particle hydrodynamics (sph) method, e.g., [145,146,147]. China has experienced sustained economic growth over the last 30 years, allowing broad knowledge development in various academic fields [148]. 

In Italy, as the second country with more contributions in the analyzed topic, representative authors such as Guzzetti F., Cuomo S., Cascini L., Sorbino G., Crosta G.B. present studies focused on numerical modelling, the application of sph and GEOtop-FS, run-out analysis and trigger factors in shallow landslides and debris flows [117,118,119,149,150]. Japan is the third country with a scientific contribution, with authors such as Imaizumi F., Sassa K., Wuang G. who highlight the effects of landslides and shallow landslides as a consequence of deforestation, groundwater flow, earthquakes, rainfall and flow path [151,152,153,154,155]. Other countries contributing in this area can be observed in Figure 5.

### 3.2. Bibliometric Mapping Analysis

The construction of bibliometric maps, depending on what is established in the methodology. Only articles and the English language are considered given their broad domain in various areas of knowledge [156,157].

#### 3.2.1. Co-Occurrence Author Keyword Network

This type of analysis allows visualizing the study area (its history and evolution) and its possible trends [158,159,160].

**Figure 6:**
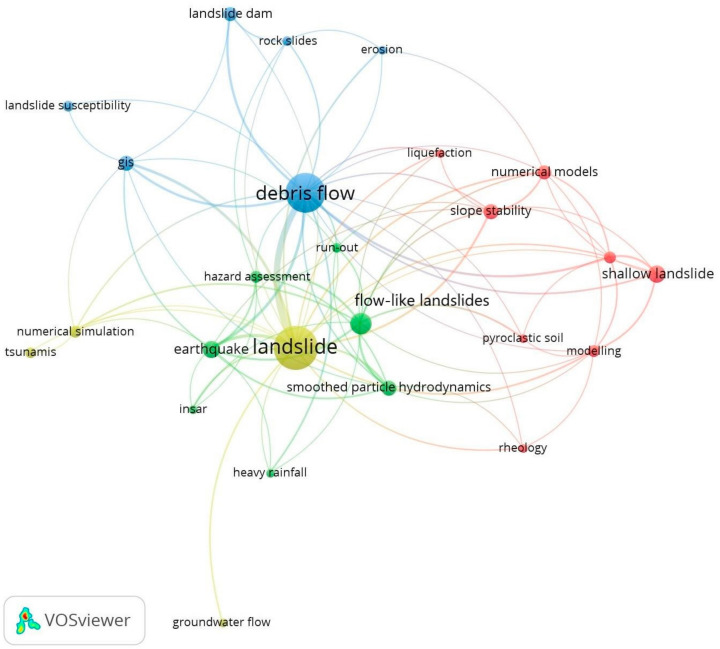
Visualization of the co-occurrence network by assigning a representative color for each cluster. Red color (shallow landslide), green color (flow like landslide), blue color (debris flow) and yellow color (landslide).

Figure 6 shows the co-occurrence network of author keywords, where 25 nodes (represents each author-keyword with at least four co-occurrences) and four clusters (groupings of nodes of the same color) are observed [161]. The figure allows a visualization of the intellectual structure of landslides to be examined in greater detail.

Cluster 1 (red color) shows studies of landslides caused by precipitation and pore pressure in the subsoil studied, due to the topography and water flow caused by rainfall [94,115,162,163,164]. These studies were carried out based on: (i) post-failure in deposits of colluvial, weathered and pyroclastic origin [118]; (ii) simulation of the probability of occurrence in hydrographic basins using GEOtop-FS [117]; (iii) the quantification of morphology and hydrological conditions [165]; and (iv) an evaluation of susceptibility and slope stability for landslide prevention [166]. Other studies reflect the slope instability that can cause significant hazards, mainly influenced by the deposit type, the rapid flows generated by seismic movements [167,168,169], large-scale deforestation [170], groundwater fluctuation, and different triggering scenarios [132,171].

Studies focusing on this cluster have led to improved mapping, understanding, interpretation and prediction of landslides, such as the movement direction through the hydraulic gradient [172], the influence of rainfall, soil saturation [125,173] and continuous monitoring for preventive decisions in potential hazardous landslides [174]. 

Cluster 2 (green color) focuses on landslides with a non-Newtonian flow behavior, demonstrated through numerical modelling, geological study and its geodynamic behavior [121,175,176,177]. These movements and trajectories are influenced by different factors such as: (i) rheology and topography [139]; (ii) hydrometeorological events such as heavy rainfall [113,178]; (iii) soil saturation in gravelly and sandy materials [178]; (iv) pore pressure impact caused by earthquakes [155,179,180]; and (v) the frontal plowing phenomenon [140]. These landslides have a natural, rapid and irregular behavior with devastating dynamics. This cluster provides the scientific community with resources to understand flow-like landslides through numerical and 3D models [181]. Models considering the smoothed particle hydrodynamics (SPH) [77,182,183,184] and the use of satellite images using methods such as InSAR [185,186,187]. These studies have allowed the modelling of submarine landslides [188,189] and landslides in landfills caused by seismic action [182]. In addition, they facilitate the affected area mapping and evaluate the intensity of the danger for the planning of adequate risk management [190]. 

Cluster 3 (blue color), these landslides can be generated by: (i) earth rubble and intense added rainfall [131,191] or when they come in contact with the mainstream [116]; (ii) failures in the landslide dam [192,193]; and (iii) the material traction on a slope, liquefaction or even due to temperature changes [105]. For its understanding, various experiments were carried out, such as the use of differential equations for the dynamics of the system [129], analysis of the theory of the critical state in the mobilization of debris flows due to the increase in the basal pressure of pores [194], and the generation of dynamic models to understand the evolution of the system [112]. For a further understanding of debris flow, maps used that are supported by Geographic Information Systems (GIS) [195,196], geophysical studies [197] and statistical methods such as logistic regression (LR) [198,199] and Multivariate Adaptive Regression Splines (MARS) were explored [200], allowing us to understand the formation or prevention of landslide dams [201,202,203] and debris flows, which can also be generated by shallow landslides, which are identified through susceptibility mapping [124,204,205].

Cluster 4 (yellow color), covers the topics written in other clusters given its great diversity or classification [36]. Its studies focus on numerical simulations for the understanding and prediction of landslides [206,207,208], which allows an understanding of the groundwater flow affectation [209,210], the infiltration of water by rainfall [211,212] and wave propagation (tsunamis) due to the collapse of slopes in bodies of water [181,213]. Recently, scientific contributions regarding landslides have been present. Multiphase flow models present submarine landslides, especially on the type and size of particles (rheology) [188]. Regarding groundwater or what is percolated by high rainfall, it is considered in Critical Rainfall Threshold (CRT) analysis, monitoring system by video camera systems and the generation of two-dimensional mathematical models by the finite difference method [214,215,216].

#### 3.2.2. Co-Citation Analysis

Co-citation analysis is one of the most widely used methods in bibliometric analysis [41]. It allows us to explore the relationships between documents, to know the knowledge base and the intellectual structure of a field of study [217,218]. Co-citation analyzes the number of times two documents are co-cited by another subsequent document [79]. When frequently cited in other publications, documents show a close relationship, which allows us to consider that they belong to the same field of research [219,220]. However, this relevance does not imply that the ideas shared by the various authors coincide with each other [221].

In this work, two co-citation methods are used: author co-citation analysis and Journal co-citation analysis, which are presented below:

##### Author Co-Citation Analysis (ACA)

This analysis is an adaptation of work by H. Small [79], done by White and Griffith [222] using the authors of the papers. ACA considers that by citing two authors more frequently in several papers, it is very likely that their fields of research are similar [223]. This makes it possible to discover the co-citation groups of reference authors that make up the knowledge base of the intellectual structure studied [73,224]. Furthermore, it allows the discovery of the academic community linked to confirming this knowledge base [225].

**Figure 7:**
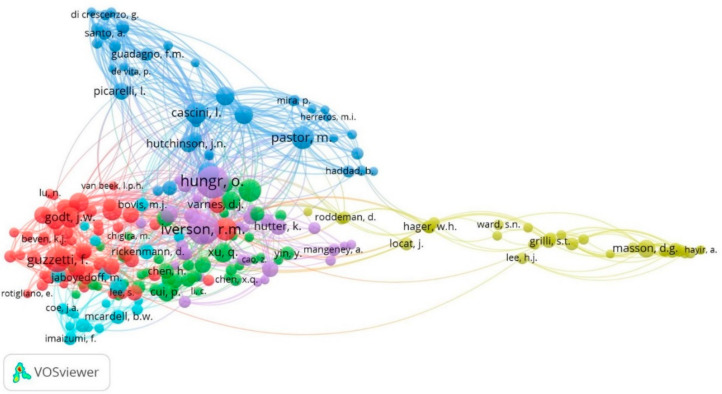
Visualization of the co-citation network assigning a representative color for each cluster. according to the number of interconnected authors. Red, green, blue, yellow, purple and light blue (in order of highest importance by VOSviewer software version 1.6.17).

Figure 7 shows this co-citation network of authors. Its construction is carried out with the VOSviewer software version 1.6.17, which uses a proprietary technique called VOS to allow a grouping of the units of analysis using similarities [74]. The nodes represent the authors’ names, which may represent topics, schools of thought or specialties [226]. The structure presents six clusters, with 235 authors possessing more than 20 co-citations.

Cluster 1 (red color) consists of 60 authors. The studies in this cluster focus on the research area of shallow landslides and debris flow influenced by rainfall or hydrological triggers [227,228,229]. These authors include Guzzetti F. (157 co-citations), in studies related to precipitation and shallow landslides [113,230]; Crosta G.B. (128) in numerical modelling and debris flow [231,232]; and Godt J.W. (107), in map generation and modelling of shallow landslides for landslide risk prevention and assessment [233,234].

Cluster 2 (green color) has 44 authors. This cluster has studies focused on the internal mechanics of landslides and debris flows, and the factors that affect the movement or detachment of material [235,236,237,238,239], in addition, it considers the run-out analysis of rock and soil slides [121,240,241]. These research topics are cover by various authors such as Sassa K., Xu Q and Wang G. with 131, 97 and 90 citations.

Cluster 3 (blue color) consists of 39 authors, some of the authors, such as: Pastor M. (126), consider the stabilization of slopes using models [119,242,243,244], while Cascini L. (122) and Evans S.G. (115), focus on modelling and studies regarding debris flow [245,246,247,248,249,250]. 

Cluster 4 (yellow color) is distant from the rest of the clusters, located at the extreme right of Figure 7. This cluster comprises 37 authors, such as Masson D.G. (79 co-citations) and his studies in the underwater landslides are influenced by groundwater [251,252,253]. Grilli S.T. (49) and Hager W.H. (46) focus on the generation of modelling and numerical simulations linked to the movement of underwater masses and subsequent tsunamis [254,255,256].

Cluster 5 (purple color) is in the central part of the structure and has 32 authors, such as Hungr O. (259), who researches runout analysis and the generation of models for risk assessment [257,258,259]. Iverson R.M. (248) and Reid M.E. (77) focused on the study of debris flow and hydrological factors such as groundwater hydraulics [260,261,262]. 

Cluster 6 (light blue color) has 23 authors, such as Takahashi T. (73), Rickenmann D. (61) and Sidle R.C. (61), where the topics of interest highlight the study and analysis of debris flow [263,264,265].

##### Journal Co-Citation Analysis (JCA)

This analysis considers the relevance and similarity of journals in a field of study to reveal the intellectual structure [225,266]. JCA studies the number of times two journals are co-cited by another journal, revealing the various research fields that make up the intellectual structure [67,267].

**Figure 8:**
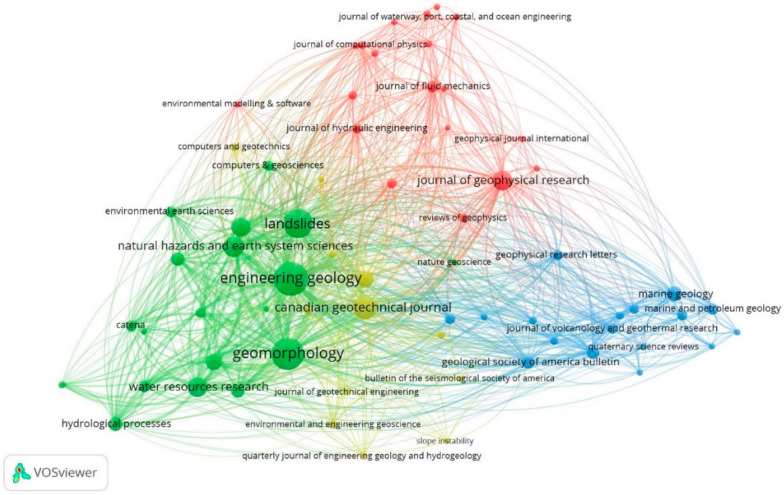
Visualization of the co-citation network assigning a representative color for each cluster (topics) and nodes (journals). According to the structure built using the VOSviewer software version 1.6.17. The colors red, green, blue and yellow appear in order of importance.

Figure 8 shows this co-citation network of journals. The VOSviewer software version 1.6.17 is used to construct and visualize the connections between the various journals represented by nodes. This network shows 69 journals with at least 20 co-citations displayed in four clusters.

Cluster 1 of red color consists of 20 journals with 1239 citations, in which the following stand out: “Journal of Geophysical Research” in the category of Agricultural and Biological Sciences, Earth and Planetary Sciences, and Environmental Science; the “Journal of Fluid Mechanics” in Physics and Astronomy; and the “Journal of Hydraulic Engineering” in Environmental Science. The latter converge in the category of Engineering.

Cluster 2 (green color) contains 20 journals and 3526 citations, focusing mainly on the category of Earth and Planetary Sciences, such as the journals of: “Engineering Geology”, “Geomorphology” and “Landslides”.

Cluster 3 (blue color) focuses on the Earth and Planetary Sciences category and consists of 17 journals with 622 citations such as: “Marine Geology”, “Geological Society of America Bulletin” and “Geology”.

Cluster 4 (yellow color) has 12 journals and 834 citations, such as “Canadian Geotechnical Journal”, in the Engineering category, and “Environmental and Engineering Geoscience”, which have a focus on Environmental Sciences. These are intertwined with the “Geotechnique” journal in the Earth and Planetary Sciences category, reflecting the interconnection with the other clusters in Figure 8.

## 4. Discussion

This study shows a consistent increase in scientific research on a landslide, thanks to the contribution of 64 countries spread over five continents (Figure 5), in 15 languages, mostly in scientific articles and in the English language. 

During the 90s, scientific production entered an introductory period, where Iverson R.M., Crosta G., and other authors contributed to the scientific community with the results of their analyses and studies (theoretical, laboratory and field) on the dynamic behavior of debris flows and landslides [101,105]. According to the Scopus database, this scientific production has experienced considerable growth since 2001 (representing 90.2% of publications).

In the decade 2001–2010, scientific research increased (Figure 3), prioritizing the update of old studies such as the global rainfall threshold [113], the classification of landslides [109] and the generation of models [117,119], which in this period are essential for understanding and preventing landslides. Over the last decade (2011–2020), the increase in its scientific production has been stable, improving the development and combination of models generated in the previous period [125,126]. In this way, the analysis of landslides and the dynamic behavior of the debris flow, shallow landslides and their movement as a flow was perfected (Figure 6).

The analysis of the intellectual structure of this field of study is conducted through three scientific maps: 

In the analysis of co-occurrence of authors keywords, the application of geographic information systems (gis) and numerical simulations are a means for the study and analysis of landslides, debris flow and flow-like landslides, e.g., [184,213]. The sph (smoothed particle hydrodynamic) method is also part of this type of analysis, in conjunction with implementing sector rheology, e.g., [149]. Numerical models are the most common method for analyzing the main issues in each cluster, focusing on modelling, erosion, slope stabilization and rainfall among others, for such study, e.g., [174].

Secondly, the author co-citation analysis allows an observation of the interconnections that the various authors have in the entire landslide field (Figure 7), which has international collaboration mainly from countries in Asia, Europe and North America (Figure 5). One of the main topics of study is the shallow landslides, which since 1988 has focused on the analysis of propagation and transformation in debris flows [268]. This issue is related to the duration and intensity of rainfall analyzed by Guzzetti, et al., (2008) [113]. The authors characteristic of this analysis, such as Sassa (green cluster), Hungr (purple cluster), Takahashi (sky cluster), Guzzetti (red cluster), among others (Figure 7), focus on the main hydrological and hydraulic, seismic and geomechanical factors causing the shallow landslide, debris flow, and consequently, the development of numerical models for risk prevention and assessment [229,232,234,235,238,241,264,265,269]. These topics are related to the red and blue clusters in Figure 6.

In addition, the existence of small groups that are isolated from those previously mentioned is observed, which we detail below: (a) the group of Pastor, Cascini and Evans (blue cluster, Figure 7), they analyzed issues related to landslide dams, erosion, the susceptibility and stabilization of slopes referring to debris flows (blue cluster, Figure 6) [244,250], which is done through simulations [243,245] and mathematical models (e.g., smoothed-particle hydrodynamics—SHP [119,245]). (b) Masson, Grilli and Hager’s group (yellow cluster, Figure 6) study the action of groundwater and its influence on mass movement (underwater and on the surface), which can trigger the generation of tsunamis or the propagation of landslides such as flows, which can be analyzed using models and numerical simulations [251,254,255,256]. These topics are closely related to the green and yellow clusters (Figure 6).

Third, in the journal co-citation analysis (Figure 8), the red cluster is observed with a broad domain about the rest of the clusters in the categories of: Engineering, Agricultural and Biological Sciences, Physics and Astronomy, Earth and Planetary Sciences, and Environmental Science. Another field of study is that of Earth and Planetary Sciences (green and blue cluster, Figure 8), focusing on the hydraulic and geotechnical properties of the material and its formation environment (geological and geomorphological) [270,271,272]. The green and blue clusters are intertwined with the yellow cluster (Earth and Planetary Sciences, Figure 8), focusing on understanding landslides, improving the models in the assessment, and their classification [273,274,275]. Instead, given the diversity of the landslide science representing the red cluster (Figure 8), it focuses on the behavior of the landslide, similar to that of a flow and the engineering analysis of the mechanical and hydraulic characteristics of the material [276,277,278,279,280]. This study is related to the group of authors Masson, Grilli and Hager (yellow cluster, Figure 7).

In this way, the entire intellectual structure and its topics of interest are analyzed, such as shallow landslide, debris flow, landslide and flow like landslide (Figure 4), which cover the five classifications made by the USGS (fall, topple, slide, spread, and flow) (Figure 1) [36].

## 5. Conclusions

This work analyses the scientific production of the research field of landslides, according to the classification addressed by the USGS. It allows an exploration and analysis of the intellectual structure of 632 publications from the Scopus database, which is feasible for a bibliometric study. When performing the performance analysis, its constant evolution is visualized between 1952–2020 (Figure 3), with a significant increase in the last 20 years. The 74% corresponds to scientific articles (Figure 4), the majority of which are in English. The scientific contribution is concentrated in 64 countries, led by China (Figure 5).

The debris flow is a type of landslide generated by various causes, such as precipitation and collapse of landslide dams. This field of study analyzes the material’s hydraulics, geodynamics and geological properties in the face of hydrometeorological and seismic events, which are an essential part of the propagation of landslides with a flow behavior and subsequent generation of debris flow (Figure 6). Some authors present studies related to the subject, such as Guzzetti F., Crosta G.B., Godt J.W., Sassa K. and Wang G., among others (see Figure 7).

The shallow landslide is an area of study supported since 1980 by Nel Caine and by Guzzetti et al., 2008, who analyze this type of landslides as a consequence of the duration and intensity of rains. This research area is in a period of growth. Therefore, it links the material’s hydrological processes and hydraulic conditions as its main triggering factors. Therefore, the implementation of numerical models for slope stabilization and risk prevention enhances their importance (Figure 6). In addition, the group of co-cited authors, such as Guzzetti, Crosta and Godt (red cluster, Figure 7), analyze a large part of these landslides, which may be the basis for understanding debris flow formation and other types of landslide.

It is essential to mention that the intellectual structure of this research field made it possible to point out or list topics of interest that can increase scientific knowledge of this subject, such as:

- The analysis of the hydraulic properties and the circumstances by which landslides can be generated as a flow;
- a deeper analysis in the study of shallow landslides and their propagation in debris flow and flow-like landslides;
- analysis of landslides from the point of view of rheology, focusing on the movement of materials caused by earthquakes and rainfalls, among others;
- generation of models through the Smoothed-Particle Hydrodynamics (SPH) method, which has been widely used for cases such as debris flow, shallow landslides, and other types of mass movements such as flows;
- implementation of satellite images in the areas of the different landslides, where the most widely implemented methods are: Interferometric Synthetic Aperture Radar (InSAR), Unmanned Aerial Vehicle (UAV), and Geographic Information System (GIS);
- stabilization studies in landslide dams, which can be caused by rainfalls and subsequent generation of debris flow;
- a technical and geological analysis on topics related to submarine landslides, among which run-out analysis and the propagation of tsunamis due to landslides and earthquakes stand out, this being an area of study that is evolving.

We consider that this study is a contribution to the academic literature due to: (i) The possibility of getting to know different researchers in specific topics of this field of study, which allows the establishment of collaboration networks; (ii) to know the experiences validated by the different authors, using techniques and methods of study that enrich scientific knowledge; and (iii) the study serves as a guide for novice researchers who wish to know in brief outlines this general structure of knowledge.

Finally, there are some limitations to this work: (a) restriction due to the classification of landslides, only to the contribution of the USGS; and (b) the use of the database (Scopus), without considering other existing bases in the academic world such as the Web of Science or Dimensions. Considering these limitations, future research is estimated using different databases and other classifications related to landslides.

## Data Availability

Not applicable.
